# Organizational Health Literacy as a Tool for Health Equity: Application in a High-Risk Infant Follow-Up Program

**DOI:** 10.3390/children10101658

**Published:** 2023-10-06

**Authors:** Lindsay E. Rosenfeld, Kelly McCullagh, Carolyn J. King, Micaela Torres, Jonathan S. Litt

**Affiliations:** 1 Harvard T.H. Chan School of Public Health, Boston, MA 02115, USA; jlitt@bidmc.harvard.edu; 2 Boston Children’s Hospital, Boston, MA 02115, USA; kelly.mccullagh@childrens.harvard.edu (K.M.); carolynjaneking@gmail.com (C.J.K.); 3 Heller School for Social Policy & Management, Brandeis University, Waltham, MA 02453, USA; 4 Charles R. Drew, UCLA Medical Education Program, Los Angeles, CA 90059, USA; micaelasierratorres@gmail.com; 5 UCLA David Geffen School of Medicine, Los Angeles, CA 90095, USA; 6 Beth Israel Deaconess Medical Center, Boston, MA 02215, USA

**Keywords:** organizational health literacy, health literacy, health equity, Healthy People 2030, assessment, NICU, early childhood development, follow-up care, follow-up clinic, high-risk infants, prematurity

## Abstract

Background: Healthy People 2030 emphasizes personal health literacy (individual skills) and organizational health literacy—the degree to which organizations equitably enable individuals to find, understand, and use information and services to inform health-related decisions and actions for themselves and others. However, research on the latter is in the early stages. Methods: This study describes an organizational health literacy assessment in a U.S. urban academic children’s hospital. A variety of evidence-based health literacy assessments were used to assess patient information materials and the environment, including institutional practices, navigation, culture and language, and communication. Trained interviewers and analysts reached consensus for all assessments. Results: *Information Items:* SMOG scores (*n* = 9) ranged from 7th- to 14th-grade reading level (average = 11.3). PEMAT-P scores (*n* = 9) ranged from 43.8% to 93.8% understandability and 0% to 80% actionability. CDC CCI scores (literacy demand) (*n* = 6) ranged from 18.2% to 58.8% (≥90% = excellent). SAM scores (suitability) (*n* = 6) fell in the “adequate” range (43.2–58.3%). The PMOSE/IKIRSCH scores (complexity) (*n* = 3) noted low-moderate difficulty. Apter’s Hierarchy (*n* = 4) revealed three numeracy domains (50% = descriptive purposes and decision-making, 100% = interpreting information). *Organization-level:* Walking interviews highlighted organizational facilitators and barriers related to the pre-visit and visit environments. HLE2 domain scores ranged from 52% to 68%. Conclusions: Organizational health literacy demands far outweigh the average literacy skills of adults in the U.S. (patients and staff). Findings can be used to hone quality improvement and other processes to focus on structural solutions to increase health equity.

## 1. Introduction

Attaining optimal health is exceptionally challenging. Many social and structural factors impede this goal, including structural racism [1,2,3,4]. Nevertheless, medicine and public health strive to promote good health even within systems that traditionally leave navigating health and related systems (e.g., housing, education) to individuals [5,6]. This happens in the context of systems that consistently demand literacy, navigation, and other skills and resources that the average person in the United States does not possess [7]. Health inequities are most certainly exacerbated by these demands [7,8].

At least 88 percent of adults in the U.S. do not have the health literacy skills necessary to navigate the healthcare system and promote their well-being [7]. This mismatch between individual skills and healthcare system demands results in poor individual health outcomes, inefficiencies in healthcare services, and increased healthcare costs [9,10,11,12]. Poor organizational health literacy is another structural barrier that exacerbates child health inequities defined by systems barriers (e.g., racism, ableism, sexism, racial residential segregation) that largely persist across time [13]. The impact of such inequities may be magnified for those born prematurely as they face an increased risk for health and developmental challenges compared to their term-born peers [14]. Therefore, guided by Healthy People 2030 (a decennial federal document outlining the nation’s public health goals), healthcare and public health are renewing their focus on reducing the burden for patients and families as they navigate health and related systems [15]. Entities like The Centers for Disease Control and Prevention and The Joint Commission, among others, have called for a prioritization of health literacy practice and research that focuses on removing barriers to accessing, understanding, and using information and services [16,17]. In addition, recent grant awards have reinvigorated the focus on health literacy, particularly at the organizational level [18].

Moreover, Healthy People 2030 emphasizes the importance of the “social determinants of health”, particularly the role of health literacy [19]. Critically, there is now a codified distinction between personal health literacy (individual skills) and organizational health literacy, an organization’s role in equitably enabling people to find, understand, and use health information. Health professionals and organizations can and certainly should partner with the education system to promote improved literacy skills and health education. Yet the most salient opportunity for medicine and public health is to carefully review and create health information, interactions, and systems that reduce the burden on patients and families. This can optimize participation and decision-making, particularly when made in collaboration with patients and families.

Organizational health literacy assessment is in its early research and practice stages overall, particularly in pediatrics. Most prior work in health and public health has focused on the relationship between low parent/caregiver health literacy and poor child health outcomes [20,21,22,23,24]. Previous research has explored the mismatch between available information and parent/caregiver skills [25,26,27]. Some attention has been given to the health literacy skills of children [28,29,30]. Still less emphasis has been dedicated to the organizational health literacy aspects of children’s health, that is, how organizational structures and processes impose barriers to optimal and equitable health outcomes [22,31,32,33].

Some research has demonstrated the importance of such assessment for organizational change to promote health equity [12,34,35]. The approach can incorporate multiple relevant systems frameworks to focus on organizational change, thereby reducing patient burden [31]. Such an assessment does not take a punitive approach. Rather, the results are used to determine the next steps in strategic planning and action, including short- and long-term decisions about improving equity in practice.

Given this, we performed an organizational health literacy assessment in a high-risk infant follow-up program at an urban children’s hospital. Such an assessment involves exploring health and health-related information and environments (e.g., institutional or organizational settings) to determine where patient and family burden can be reduced (i.e., ease of understanding and performing of health-related tasks or actions). Assessment produces data that can suggest changes to materials, interactions, websites, signage, processes, and spaces in clinics, hospitals, and beyond [12,36,37,38,39].

In this program, high-risk infants are followed from Neonatal Intensive Care Unit (NICU) discharge through three-years-old to monitor and intervene in relevant medical and developmental care. Families are referred to the program by providers from any hospital, and engagement in the program is specialized based on the child’s needs. Participation in follow-up programs is important for several reasons. Infants needing intensive care after birth due to prematurity or significant illness are at risk for ongoing health and developmental challenges over time [14]. Many have a significant need for medical care and developmental support in infancy and early childhood [40]. And despite it being the recommended model of pediatric care, preterm infants often lack a family-centered medical home [41]. High-risk infant follow-up programs provide medical and developmental surveillance, referral to needed subspecialty care and therapeutic services, and assistance with care coordination [42]. Despite this, inequities in program participation often reflect structural barriers that impose obstacles by family race, language, and neighborhood. Infant follow-up program participation has been linked to such factors [43,44]. We explored the organizational health literacy environment as a modifiable cause of potential barriers to optimal care and well-being in this high-risk population. As such, we studied the literacy and other demands placed on families that engage with, or try to engage with, the infant follow-up program. We also investigated what opportunities exist for the program to make changes to better meet the skills of its families.

## 2. Materials and Methods

We conducted an organizational health literacy assessment in a clinic program housed at an urban U.S. academic children’s hospital. We followed the Consolidated Criteria for Reporting Quality Research guidelines for best practice reporting of qualitative results [45]. Our core research team included a social epidemiologist (LER), a neonatologist (JL), and neonatal-perinatal medicine fellow (KM), and two public health graduate students (MT, CJK). All information and environments assessed were public.

### 2.1. Information Items

For analysis, clinic staff identified nine frequently used patient-facing items or materials. (Table 1) Items included patient education resources (*n* = 4), intake forms (*n* = 2), and clinic visit or referral documents (*n* = 3). Of these, four were found on the clinic website, and five were print materials recommended to study staff by the clinic. None of the items were videos, social media, or audio, as the clinic does not use these media.

### 2.2. Environment Locations

The clinic operates as a specialized program of a large, quaternary care children’s hospital. The program serves about 400 families annually and provides medical consultation, assessments of cognitive, language, social, behavioral, and motor development, and family psychosocial and material needs. During the assessment period, the clinic changed locations from the main hospital to a satellite location < 1 mile away. As such, three assessments (2021) were conducted in the new location, and one assessment (2019) was conducted in the old location. Families still frequent the old location (the main hospital) for related appointments. A second, or satellite, clinic location was not included because it was not operating when the assessment began. No in-person environmental assessments were conducted in 2020 due to restrictions posed by the COVID-19 pandemic.

### 2.3. Data Collection

#### 2.3.1. Information Assessments

To identify literacy demand, six standardized assessment tools were used to evaluate the information items. Each one offers an analysis of a different component of literacy demand. A total of 42 assessments were performed overall across nine information items. (Table 2) The assessments used were:

Simple Measure of Gobbledygook (SMOG): Readability—The SMOG assesses reading grade level and represents the grade-level standard skills needed to comprehend and use the item [46].

Suitability Assessment of Materials (SAM): Suitability—The SAM examines six components of documents or videos: content, literacy demands, graphics, layout, typography, learning stimulation, motivation, and cultural appropriateness [47].

Peter Mosenthal and Irwin Kirsch readability formula (PMOSE/IKIRSCH): Complexity—The PMOSE/IKIRSCH evaluates document complexity, i.e., the structure, organization, and density of tables and charts within a document or video. Results describe the reading level, material complexity (very low to high), and proficiency demands [48].

Patient Education Materials Assessment Tool for print material (PEMAT-P): Understandability and Actionability—The PEMAT-P assesses understandability (information comprehension) and actionability (action assistance) of print items by evaluating components such as content, word choice and style, numbers, organization, layout and design, visuals, and ability to activate [49].

Centers for Disease Control and Prevention Clear Communication Index: Suitability—The CDC CCI provides a comprehensive literacy demand score of a document or video by assessing the main content and organization, behavior, numbers, and risk [50].

Apter’s Numeracy Hierarchy (hereafter, the Hierarchy): Numeric Demand—The Hierarchy assesses documents’ demands related to numerical concepts. The Hierarchy helps to identify whether readers must describe (low demand), interpret (medium demand), or make a decision (high demand) based on the numerical information presented [51].

#### 2.3.2. Environmental Assessments

The Health Literacy Environment Activity Packet (HLEAP), First Impressions, and Walking Interview were used to assess the clinic environment [52]. In this assessment, two participants engaged in step-by-step questions for three structured activities focused on calling the institution, visiting the website, and walking to the entrance [12]. The participants continue observations in a “navigation visit” comprised of six stages: (1) Observations at the Entry Point or Lobby (What is the overall literacy environment?), (2) Directions/Seeking Help (Is help with navigation available?), (3) Navigation (What is it like to navigate to a specific location?), (4) Observation (What are the literacy demands or assumptions patients encounter as they access services?), (5) Reflections (What is the overall impression, e.g., use of the written word, navigation aids, signs, language?), and (6) Feedback/Next Steps (What was learned? What are the next steps?). Open-ended prompts throughout the process gather impressions, identifying barriers and facilitators. HLEAP assessment focused on the main hospital (*n* = 1) and the primary clinic location (*n* = 3). The secondary clinic location was not included, yet the phone and website pertained to any physical location of the clinic.

In addition, the Health Literacy Environment of Hospitals and Health Centers (HLE2) was used to conduct a detailed evaluation in four domains: (1) Institutional Practices, (2) Navigation, (3) Culture and Language, and (4) Communication. The full HLE2 also includes a first section examining organizational policies. However, this was omitted since this study focused on clinic-level practices and experiences that are within the clinic’s oversight. LER and JSL conducted the HLE2 in consultation with KM, CJK, MT, and the clinic coordinator (CC, see acknowledgments). The HLE2 assessment also focused on the primary clinic location only. The HLE2 assessment began at the main hospital site but was interrupted by the start of the COVID-19 pandemic. When assessment resumed, the clinic had already moved to its new location, and the HLE2 assessment was performed solely for the new primary location.

### 2.4. Data Analysis

#### 2.4.1. Information Assessments

The content, layout, and type of an item determined the tool used. Time and funding also drove the number of tools used. For instance, the PMOSE-IKIRSCH can only be used for items containing charts or tables, and the Hierarchy can only be used if numbers or numerical concepts are present. Prior to analysis, and based on these criteria, LER, CJK, and MT came to a consensus on the appropriate tools for each item. The PMOSE/IKIRSCH was used to assess a table or chart (*n* = 3), and the Hierarchy explored the demand of numerical concepts (*n* = 4). All items were assessed with SMOG (*n* = 9) and PEMAT-P (*n* = 9); some items were assessed with the SAM (*n* = 6) and CDC CCI (*n* = 6) to further elucidate the literacy demand and appropriateness of the written material for the target audience. Percentages for SAM, PEMAT, and CDC CCI scores were calculated by dividing the score by the total possible points applied for each assessment.

Two different SMOG scores were calculated for five materials. The first score was computed by following the directions exactly as written, while the second computation (modified SMOG) counted a repeated polysyllabic term or phrase only once. For example, for the Retinopathy of Prematurity Overview, a SMOG score was calculated with and without the phrase “retinopathy of prematurity” to reveal what impact the repetition had on the final score, if any. A numeracy assessment of the complexity and comprehension of relevant materials was conducted following Apter’s model [51], whereby numerical demand—describe, interpret, or decision-making—was used to evaluate the numerical components of the item.

Two coders (CJK, MT) separately completed each assessment for each item. Then, they met to discuss any discrepancies and reach a consensus on all assessments for all items. Any discrepancies were resolved, and no third coder was needed. LER did a random quality check of assessments across items (~20%) and consistently agreed with coders’ consensus scoring [53,54].

**Table 2:** Health literacy assessment scores by item.

	Assessment Tool						
	SMOG *	PMOSE/ IKIRSCH	SAM	CDC CCI	PEMAT Understandability	PEMAT Actionability	Numeracy ^^^
**Print Material**							
**Family Feedback Form**	12	Low Level 2 Grade 8	-	-	11/17 (64.7%)	4/6 (66.7%)	-
**Clinic Welcome Packet**	14 (13) ^1^	Very Low Level 1 Grade 5	22/44 (50%)	10/17 (58.8%)	10/15 (66.7%)	2/5 (40%)	✓
**MCHAT-R**	8 (6) ^2^	-	-	-	7/11 (63.6%)	3/5 (60%)	-
**ASQ-42 Month**	7	Moderate Level 3 Grade 9	-	-	15/16 (93.8%)	4/5 (80%)	-
**Turning Three**	11	-	19/44 (43.2%)	6/18 (33.3%)	7/16 (43.8%)	3/6 (50%)	✓
***Average Score:***	*10.4* *−9.5*	*Low* *Level 2* *Grade 8*	*46.6%*	*46.1%*	*66.5%*	*59.3%*	*-*
**Website Material (clinic)**							
**Retinopathy of Prematurity Overview**	14 (13) ^3^	-	16/36 (44.4%)	3/11 (27.3%)	10/13 (76.9%)	0/0 (0%)	✓
**Hearing Loss Overview**	12	-	16/36 (44.4%)	4/11 (36.4%)	7/12 (58.3%)	1/5 (20%)	✓
**Cerebral Palsy Overview**	13 (12) ^4^	-	16/36 (44.4%)	2/11 (18.2%)	8/12 (66.7%)	0/0 (0%)	-
**Your Visit**	11 (11) ^5^	-	21/36 (58.3%)	6/17 (35.3%)	9/13 (69.2%)	2/5 (40%)	-
***Average Score: Website***	*12.5* *−12*	*-*	*47.9%*	*29.3%*	*67.8%*	*15%*	*-*
***Average Score: Total*** ***(Print & Website)***	*11.3* *−11*	*Low* *Level 2 Grade 8 (print)*	*47.5%*	*34.9%*	*67.1%*	*39.6%*	*-*

* Modified SMOG: SMOG calculation *without* one repeated polysyllabic word or phrase. The modified SMOG explores whether that repeated word or phrase is driving the SMOG score. ^^^ See Table 3 for further details. ^1^ Removed: clinic name. ^2^ Removed: Example. ^3^ Removed: Retinopathy of Prematurity. ^4^ Removed: Cerebral. ^5^ Removed: clinic name.

#### 2.4.2. Environmental Assessments

HLEAP: We used thematic analysis to generate themes from the detailed responses, specifically content analysis and a grounded theory approach [55]. Graduate students enrolled in LER’s organizational health literacy course generated reports per the HLEAP guidelines, and thereafter, KM and LER conducted a thematic analysis of the reports’ contents. Codes and themes arose from the data. Analysis was stratified by the pre-visit environment (characterized by phone and website interactions that happened prior to a visit) and the visit environment (characterized by walking to the entrance, entering the lobby, and journeying to the destination). Researchers stored and organized data in Microsoft Excel. To start, two coders (KM, MT) independently reviewed each report and identified salient blocks of text. They met to come to a consensus on the relevant text. Then, the coders independently generated codes to capture salient points. Next, they conferred to reach a consensus on codes. Thereafter, they independently assigned themes to each code. Once themes were identified, the coders conferred to reach a consensus on themes. After each step, the initial coders met with a third coder (LER) to resolve any discrepancies in salient text, codes, and themes. Thematic analysis was deemed complete when all three coders reached a consensus for all codes and themes. Final themes are those that arose ≥50% of the time within or across components. Themes and data saturation were discussed with all authors to affirm face validity.

HLE2: Ahead of scoring, two scorers (LER, JSL) reviewed the HLE2 and discussed any questions about assessment prior to scoring, coming to a consensus on question interpretation or what terms mean in the clinic context. They independently scored each HLE2 item, section by section. Then, they discussed each item and came to a consensus. Score consensus was validated by KM (all sections) and YM, MT, and CJK (communication only). A scoresheet was used to compute HLE2 scores for each section. Only section and sub-section scores are produced; there is no overall HLE2 score. The scores are not weighted, and sections are scored independently from each other.

## 3. Results

### 3.1. Information Assessment

Print Items: Printed items provided to families included information packets and assessments. For print items given to patients (*n* = 5), the average SMOG score (*n* = 5) was 10.4, or more than a 10th-grade reading level demand (range: 7–14). The clinic welcome packet scored 14, or 14th-grade-level reading demand, which was the highest print item SMOG score. The average of the modified SMOG scores for print items (*n* = 2) was 9.5, or more than a 9th-grade-level reading demand. The PMOSE/IKIRSCH scores (*n* = 3) revealed very low to moderate table complexity, ranging from 5th- to 9th-grade-level demand. Two items were assessed using the SAM (average score 46.6% (adequate)). SAM criteria such as writing style and learning stimulation and motivation were considered adequate or superior, while reading grade level, relevance of illustrations, and layout were rated as not suitable. The average CDC CCI score was 46.1% (range: 33.3–58.8%) (*n* = 4). The items met the criteria in categories such as everyday language use and use of headings and chunking but did not meet the criteria in categories such as containing a main message at the beginning of a summary of important information. The average PEMAT Understandability score was 66.5% (range: 43.8–93.8%), and the average PEMAT Actionability score was 59.3% (range: 40–80%) (*n* = 5). The items met the PEMAT criteria in categories such as use of everyday language, word choice and style, calculations, and general organization, but not in categories such as use of medical terms, number understandability, and inclusion of a summary. The Hierarchy (*n* = 2) revealed two demand domains for print items: decision-making and interpreting information.

Website Items: Items assessed on the clinic website (*n* = 4) focused on patient education information (*n* = 3), with one program overview. The average SMOG score (*n* = 4) was 12.5, or more than a 12th-grade-level reading demand (range: 11–14). The average modified SMOG score (*n* = 3) was 12, or 12th-grade-level reading demand (range: 11–13%). Again, the average SAM score (*n* = 4) was adequate (average, 47.9%, range: 44.4–58.3%), with sentence construction, motivation, and cultural match rated as adequate or superior and reading grade level and content, including content about behaviors and inclusion of a summary or review, rated as not suitable. The average CDC CCI score was 29.3% (range: 18.2–36.4%) (*n* = 4). The items met the CDC CCI criteria in categories such as the use of lists and chunking but not in categories such as the use of a visual cue to support the main message, active voice, or inclusion of a summary in the first section. The average PEMAT Understandability score was 67.8% (range: 58.3–76.9%), and the average PEMAT Actionability score was 15% (range: 0–40%) (*n* = 4). The items met the PEMAT criteria in categories such as use of active voice, chunking, header, and sequence organization, but not in categories such as actions the user can take or the presence of a tangible tool. The Hierarchy (*n* = 2) revealed two demand domains for website items: descriptive purposes and interpreting information.

### 3.2. Environmental Assessment

**Figure 1:**
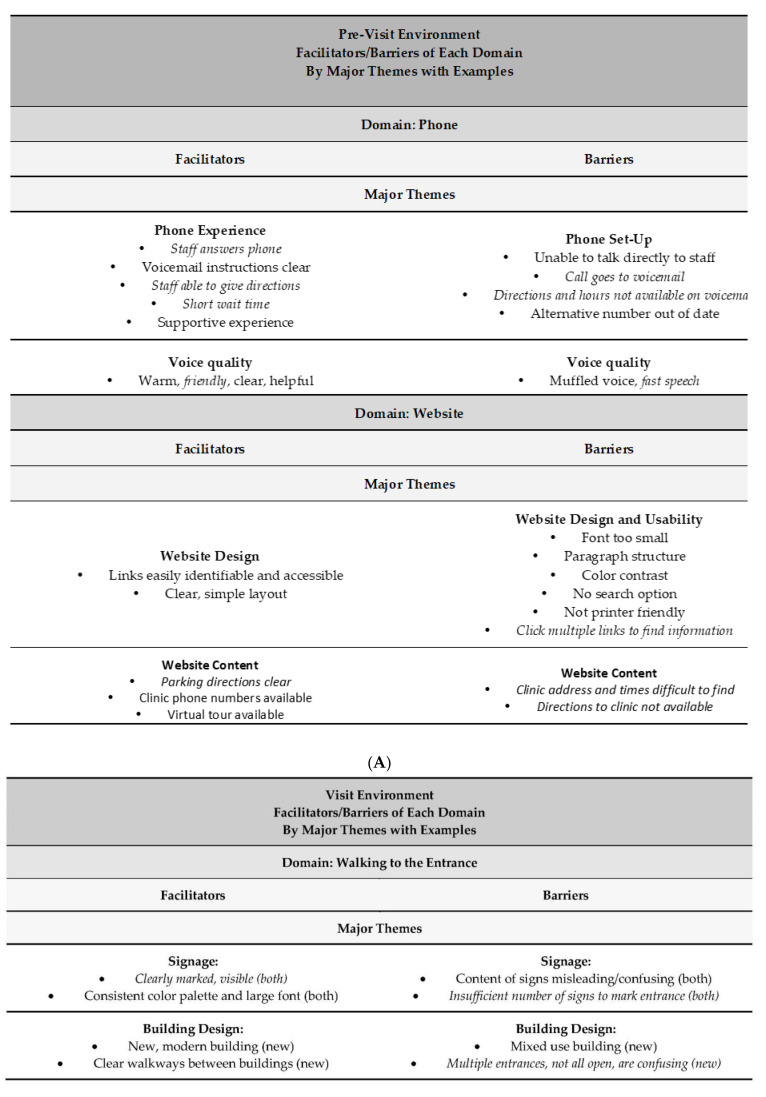

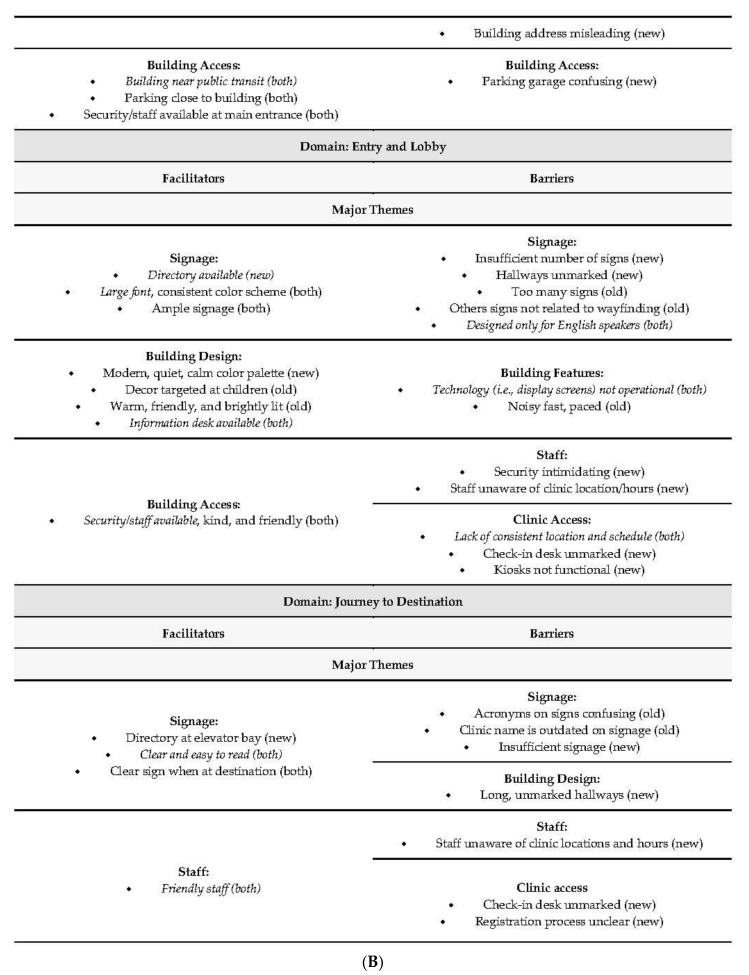
(**A**): Pre-Visit Environment. Major Themes: noted in ≥50% of assessments (≥2/4). Italicized text indicates specific examples addressed by 2 or more observers within a theme. (**B**) Visit Environment. Major Themes: noted in ≥50% of assessments (≥2/4). Italicized text indicates specific examples addressed by 2 or more observers within a theme. (old) = comment pertaining to prior clinic location. (new) = comment pertaining to new clinic location. (both) = comment pertaining to both clinic locations.

Health Literacy Environment Activity Packet: HLEAP assessment highlighted organizational facilitators and barriers. Themes were stratified by pre-visit environment (Figure 1A) and visit environment (Figure 1B), presented within and across categories. An idea was identified as a theme when at least 50% of the assessments (≥2/4) noted similar observations.

#### Pre-Visit Environment

The HLEAP pre-visit environment domains focus on the phone and website experiences prior to the actual healthcare visit.

Phone: Two themes in the phone domain were identified as facilitators: the phone experience (e.g., the staff answers phones, staff able to give directions, short wait time) and voice quality (e.g., friendly). There were also two themes identified as barriers: the phone set-up (e.g., phone goes to voicemail, directions, and hours not available on voicemail) and voice quality (e.g., fast speech).

Website: Two themes in the website domain were identified as facilitators: the website design (e.g., clear, simple layout) and website content (e.g., parking directions clear). There were also two themes identified as website barriers: the website design or usability (e.g., clicking multiple links to find information) and website content (e.g., clinic address and times difficult to find, directions to the clinic not available).

### 3.3. Visit Environment

The HLEAP visit environment section focuses on the experience of navigating to the clinic for a visit. The domains explored were: walking to the entrance, entry, and lobby, and journey to the destination. In this work, the follow-up clinic was the pre-determined destination. The main clinic changed location in 2021. Assessments (*n* = 4) spanned from 2019 to 2021. As such, one assessment was conducted in 2019 (old location at main hospital), where clinic patients still go for myriad other appointments, and three assessments were conducted in 2021 (new clinic location). Major themes were identified by location, whether in both clinic locations (“(both)” in Figure 1B), the old clinic location only (“(old)” in Figure 1B), or the new clinic location only (“(new)” in Figure 1B).

Walking to the Entrance: Three themes were identified for the walking to the entrance domain: signage, building design, and building access. As facilitators, assessment uncovered signage that was clearly marked and visible, building design that included clear walkways between buildings, and building access that revealed the building was near public transit. As barriers, the assessment revealed signage that had an insufficient number of postings to mark the entrance, building design that had multiple possible entrances, all of which were not open, which led to confusion, and building access that exposed a confusing parking garage process.

Entry and Lobby: Facilitators included signage (e.g., directory available, large font), building design (e.g., information desk available), and building access (e.g., security/staff available). Barriers included signage (e.g., designed only for English speakers), building features (e.g., technology, such as display screens, not operational), staff (e.g., security intimidating), and clinic access (e.g., lack of consistent location and schedule).

Journey to Destination: Facilitators included signage (e.g., clear and easy to read) and staff (e.g., friendly staff). Barriers included signage (e.g., insufficient signage), building design (e.g., long, unmarked hallways), staff (e.g., staff unaware of clinic locations and hours), and clinic access (e.g., registration process unclear).

Health Literacy Environment of Hospitals and Health Centers (HLE2): The average score across the main HLE2 domains was 63% (range: 52–68%). The Navigation domain scored the highest (68%), with Part 1: Arrival scoring at 22%, and Part 2: Wayfinding scoring at 89%. Navigation Part 1 sub-domain scores ranged from 0% to 22%, while Part 2 sub-domain scores ranged from 83% to 100%. Thereafter, the Institutional Practices and Culture and Language domains both scored 66%. Institutional Practices Part 1: Resources scored 100%, and Part 2: Orientation, Development, and Expectations scored 55%. Institutional Practices Part 1 had no sub-domains, while Part 2 sub-domain scores ranged from 39% to 69%. Culture and Language had no parts or sub-domains. Finally, Communication scored the lowest (52%). Sub-domains ranged from 42% to 71%; there were no parts (Table 4).

## 4. Discussion

We found that the literacy and related demands of the program surpassed the average literacy skills of U.S. adults. The program’s information and environment are not as understandable, accessible, and actionable as needed for optimal health outcomes. Improvements must be made to create equitable opportunities for all families and patients to understand, access, and use the information and services.

Information Demand: Scores varied by information type, i.e., print materials vs. websites. Overall, the average SMOG score (reading level demand) was higher for text on websites than for print materials. Whereas the average PMOSE/IKIRSCH (document complexity) and SAM (suitability) scores did not vary by material type. The average CDC CCI (suitability) scores were higher for print materials than for website items. The average PEMAT Understandability scores were almost the same for website items and print materials, whereas the PEMAT Actionability scores were higher for print materials than for website items. Thus, where there was variability, the higher scores pertained to print materials text compared to online material, indicating better readability, understandability, and actionability.

Environment Demand: The main HLE2 domain scores ranged from 52% to 68%, suggesting the need to augment efforts to eliminate literacy-related barriers across domains in the environment [56]. More nuance was evident when exploring across sub-domains and parts within sub-domains. One sub-domain (Institutional Practices, Part I: Resources) and one part within a sub-domain (e.g., Part 2: Wayfinding, Services and Specialty areas) scored 100%. This means the program affirmed the presence of resources (*n* = 5) like a staffed library/resource room for staff and patients, and the presence of clear signs/processes (*n* = 2) like clearly posted clinic name/area and clear sign-in procedures, respectively. However, other sub-domains or parts within sub-domains scored much lower, with the lowest part scoring 0% (e.g., Navigation, Part I: Arrival, Arrival and Departure), meaning the program did not provide clear postings for arrival and departure (*n* = 4); for instance, there was no clear posting of directions from the parking area to the main entry.

Overall, the HLEAP thematic results suggested simultaneous facilitators of and barriers to interacting with the program environment. Across pre-visit domains (e.g., phone, website), clear directions were a facilitator when directions were present or provided by a staff member. Yet for both domains, directions were often not present or easily located, which was identified as a barrier. Across visit domains (e.g., walking to the entrance, entry and lobby, journey to destination), signage could be a facilitator when clearly indicated (e.g., visible, large font), but also a barrier when only designed for some (e.g., English speakers).

Providers are inadequate judges of parental health literacy [57]. These results are particularly meaningful because of the significant stress experienced by parents/caregivers of high-risk infants compared to parents of term babies [58]. As such, reducing the burden of and barriers to the healthcare system is even more pertinent for attaining equitable opportunities for optimal outcomes in this population. Taken together, health and related systems can move more equitably and efficiently toward the goal of optimal service user and family outcomes by reducing the mismatch between system demand and user/family skill [59,60]. Thus, it is especially important that the information and environments are accessible and actionable for all.

### 4.1. Moving Forward

Collectively, these data demonstrate excellent practices as well as opportunities for improvement. The next steps can be best accomplished in collaboration—with leadership, among staff, and, most importantly, with families. Collaboratively, this team of stakeholders can create an organizational health literacy action plan to lay out the lessons learned and related action steps. The AHRQ Health Literacy Universal Precautions Toolkit recommends PDSA (plan–do–study–act) cycles to accelerate implementation and evaluation [61].

To that end, the program leadership team and staff are planning to convene a task force to address gaps and make improvements. The task force will use these data as a baseline from which to develop, implement, and test the effectiveness of changes to the program. Examples include revising written materials to improve readability and actionability, updating the website with current and usable information, and ensuring communication, written material, and wayfinding aids are accessible and presented in families’ preferred languages. There is an added sense of moral urgency to act given the noted racial, ethnic, and language inequities in program participation. The team is keen to see not only increased scores on measures of organizational health literacy but also improvements in communication and engagement with families to achieve optimal experiences and outcomes.

### 4.2. Lessons Reinforced

Creating accessible, understandable, and actionable information and environments is a complex and multidimensional undertaking. Organizational health literacy assessment offers a concrete multi-faceted approach, which can be adapted to the interests, needs, and capacity of an institution. It highlights what is working and offers concrete change strategies for what is not working so well. No matter how or where an organization begins, health literacy is a critical tool for collaborating across an institution, with partners and with the public in pursuit of health equity. It is crucial to remember that most families interact with health and health-related information or websites so they can better understand something (e.g., risk for developmental delay), access a service (e.g., housing vouchers), and/or make a decision (e.g., Do high-risk infants follow a typical vaccine schedule?).

### 4.3. Five Recommended Next Steps (Figure 2): These Results Suggest a Need for Evidence-Based Practice as Follows

#### 4.3.1. Prioritize Plain Language in Written Text, Conversations, and Signage

Reduce complex words and long sentences; this includes both medical and developmental jargon as well as other language. An example from our assessments, words like subsequent, approximately, comprehensive, physician, ensure, evaluate, consultation, and interdisciplinary can be eliminated from the study program information. This may help lower reading demand and improve understanding.

**Figure 2:**
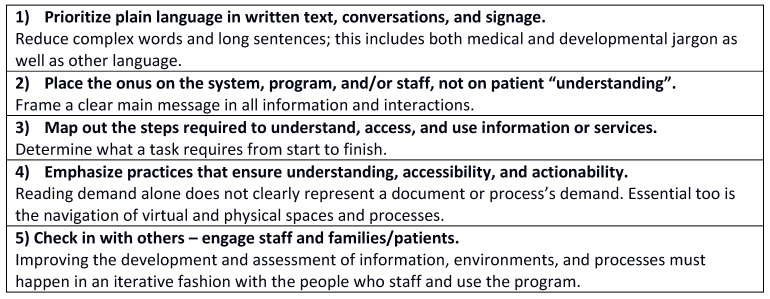
Five steps for incorporating organizational health literacy practice into your work.

#### 4.3.2. Place the Onus on the System, Program, and/or Staff, Not on Patient “Understanding” or Individual Skills

Frame a clear main message in all information and interactions. For example, items on the MCHAT-R checklist or the program’s welcome packet lacked clear main messages that frame the content, focus the reader, and create easy opportunities to make the information usable. More specifically, the information items were largely devoid of concrete, actionable instructions, or recommendations. The ultimate goal of written materials is that they are of use to the reader.

#### 4.3.3. Map Out the Steps Required to Understand, Access, and Use Information or Services

Determine what steps a task requires from start to finish. Explore the components of making an appointment and arriving for that appointment. Consider facilitators (e.g., clear signage, building design, access, staff) and challenges in making and getting to an appointment (e.g., phone set-up, voice quality, website design, usability and content, signage, building design, access, and features, clinic access). For example, multiple program locations may prove confusing for booking appointments and getting to the right place when the website is difficult to navigate, phone numbers are incorrect, contact with a live person is challenging, or check-in signage is poor. The HLE2 scores suggest that institutional practices, navigation, culture and language, and communication all play a role in the journey across required tasks.

#### 4.3.4. Emphasize Practices That Ensure Understanding, Accessibility, and Actionability

Reading demand alone does not clearly represent a document or process’s demand. No single text or website item or environment (i.e., sign, directions) in our assessment met all thresholds for readability (i.e., SMOG), complexity (i.e., PMOSE/IKIRSCH), suitability (i.e., SAM, CDC Clear Communication Index), understandability (i.e., SAM, CDC Clear Communication Index, PEMAT), accessibility (i.e., SAM, CDC Clear Communication Index), or actionability (i.e., PEMAT).

Essential too is the navigation of virtual and physical spaces and processes. For example, the program’s welcome packet asks patients/families to make an appointment but does not provide concrete action steps to do so. The themes that arose from the HLEAP assessment noted how signage, the building, and staff can facilitate and create challenges to the arrival and entry process. A frustrating experience when arriving to services sets the tone for the upcoming interaction and can even impart an unwelcome tone, suggesting that this place is not suitable for them. Friendly, present, and available staff are as important as signage that is clearly marked, visible, in large font, and easy to read. Outdated information and signage designed only for English speakers, containing outdated information, medical jargon, or acronyms, can add to the confusion.

#### 4.3.5. Check In with Others—Engage Staff and Families/Patients

Improving the development and assessment of information, environments, and processes must happen in an iterative fashion with the people who staff and use the program. Any assessment performed in this study can be completed in collaboration with staff and families. Certainly, this takes additional planning, time, funds, and coordination, which this study lacked. However, pieces of it can also be incorporated into staff training and meetings and with patients at visits or via paid family advisers. As part of this work, to be published in the future, we interviewed family, staff, and key informants about many of the issues raised in the assessments. The results highlighted the importance of each and every assessment dimension across levels. Families and staff will be involved in critical decisions as the program continues to process the next best steps.

The best evidence suggests that plain language, well-organized information, actionable steps, and clear document, website, process, and building design increase accessibility, trust, and usability [62].

### 4.4. Implications

Organizations vary in their readiness for change. Applying an organizational health literacy perspective can help organizations at all stages consider what is going well and what could be improved to help move the health equity journey forward. The status quo is an expectation that patient skills meet current system demands. An organizational health literacy assessment explores how the system can create more equitable processes and outcomes to attain health equity. This is essential because, on average, adults in the U.S. do not have the skills to sufficiently access, understand, and use information and services [63]. Trying to “fix” patients, characterizing “no shows” as disinterested, or presuming “adherence” is only possible when family care obscures the problem. Institutions, programs, and staff must engage families/patients in new questions and assessment activities that uncover what is working well and what needs to change. Organizational health literacy assessments are feasible, adaptable, and effective. They are an essential tool for developing policies, processes, and practices that can lead to equitable processes and equitable outcomes.

This is especially true for children with medical and developmental complexity, given the fragmented healthcare environment and many obstacles faced by families and caregivers. Preterm birth has a significant impact on an individual’s health in the near- and long-term. It is vital and a moral responsibility to ensure access to care in the NICU, in the transition to home and community, and throughout childhood. Changes to health systems and health services delivery must keep pace with the growing evidence of the life course implications of preterm birth for health and development. As such, the need for healthcare organizations to address health literacy generally, and perhaps particularly in “high risk” situations, is more relevant than ever [64]. It is certainly pertinent in the care of premature infants where care transitions and communication about complex care plans are standard. For almost all families, the transition from NICU to home, including outpatient pediatric services, is highly stressful.

### 4.5. Limitations

Despite extensive time and effort in applying the best methods and research practices, this study has limitations. First, all reviewed information items were selected by the clinic coordinator according to the program’s needs across three information categories required by the research team: access (e.g., making an appointment, getting to the clinic), content (e.g., program, services, health issues), and how-to/follow-up (e.g., with the clinic, appointments, medications, therapies). Therefore, there may be bias in material selection, which could skew findings in either direction. Additionally, no individual assessment fits any one item perfectly, and some items may not be fully assessed because robust tools do not exist (e.g., numeracy, document tone, racial and cultural bias). The best evidence was used to highlight these and other issues, but formal tools were not yet available. For environment assessment, it is important to keep in mind that patients were not involved and as such, there was no access to the many spaces and interactions that patients encounter in the lead-up to or during an actual visit. Finally, the HLEAP is an experiential assessment; we generated themes across four HLEAP “journeys”. Despite a grounded theory approach, the assessment questions expectedly shaped emergent themes (e.g., phone, website).

## 5. Conclusions

Building trust is crucial to ensuring that families feel welcome and at ease [62]. Ensuring that systems are health literate is one piece of the puzzle, especially because health literacy is an interaction influenced by both individual and societal contexts [65]. When systems do not meet the needs of their users, distrust festers and barriers are erected. This mismatch affects minoritized populations in the United States in greater numbers, which exacerbates health inequity. Personal health literacy varies dramatically because of individual skills driven by inequitable educational and other experiences—and yet, it changes across the life course and fluctuates in high-stakes emotional encounters, like caring for a premature infant. Yet optimal organizational health literacy can help to counteract these barriers and move us toward health equity. Pediatrics and any services devoted to children and families can use organizational health literacy as a tool for building processes and institutions that invite families into a respectful and supportive exchange that engenders trust and support. When systems equitably enable families to find, understand, and use services, these families are more equitably able to engage with those services and derive the most out of their care.

## Figures and Tables

**Table 1:** Description of Materials.

Material	Description
**Family Feedback Form (2021)**	A two-page handout given to parents/guardians of children seen in the clinic. The document outlines the major findings and recommendations from the visit and common referrals and resources.
**Clinic Welcome Packet (2021)**	A comprehensive letter distributed to parents/guardians of the clinic. It summarizes the program goals and objectives, answers common questions, and provides a referral form.
**MCHAT-R (2021)**	The Modified Checklist for Autism in Toddlers–Revised. A parent-reported screening checklist to assess the risk of an autism spectrum disorder diagnosis among young children.
**ASQ-42 Month (2021)**	The Ages & Stages Questionnaires, 42 Month. A parent-reported assessment to screen for developmental delays or other crucial milestones when a child is 42 months old. The questionnaires are completed by parents and scored by professionals.
**Clinic Website|Retinopathy of Prematurity Overview (2021)**	A web page connected to the clinic on the hospital website. It aims to educate parents/guardians on retinopathy of prematurity in children.
**Clinic Website|Hearing Loss Overview (2021)**	A web page connected to the clinic on the hospital website. It aims to educate parents/guardians on hearing loss in children.
**Clinic Website|Cerebral Palsy Overview (2021)**	A web page connected to the clinic on the hospital website. It aims to educate parents/guardians on cerebral palsy in children.
**Clinic Website|Your Visit (2021)**	A web page connected to the clinic on the hospital website. It aims to educate parents/guardians on the details of each visit to the clinic.
**Turning Three (2021)**	A presentation offered to parents/guardians of the clinic, developed by the Federation for Children with Special Needs Parent Training and Information Center, to help educate parents/guardians on the intricacies of Early Intervention and Special Education starting at age 3.

**Table 3:** Numeric Complexity Scores.

Material	Numeric Demand		
	Describe	Interpret	Decision
** Clinic Welcome Packet **	0%	33.30%	66.70%
** Clinic Website—Retinopathy of Prematurity Overview **	60%	40%	0%
** Clinic Website—Hearing Loss Overview **	40%	80%	0%
** Turning Three **	0%	33.30%	66.70%

Percentage scores calculated from the proportion of relevant numeracy elements scored for each category of numeric demand.

**Table 4:** HLE2 Scores by Domain and Sub-Domain, New Clinic Space *.

Domain **	Sub-Domain, Part	Score % ^+^
**Institutional Practices**	--	**66%**
	Part 1: Resources	100%
	Part 2: Orientation, Development,	55%
	and Expectations	
	Orientation	69%
	Development	30%
	Expectations	67%
**Navigation**	--	**68%**
	Part 1: Arrival	22%
	Arrival and Departure	0%
	Entry and Lobby Access	40%
	Part 2: Wayfinding	89%
	Staff Assistance	94%
	Hallways and Navigation Ease	83%
	Services and Specialty Areas	100%
**Culture and Language**	--	**66%**
**Communication**	--	**52%**
	Print Materials	46%
	Forms	42%
	Websites	51%
	Patient Portals	71%

* The *new clinic space* refers to the clinic in its current physical location. ** The *organizational policies* section was not completed because the focus was on the clinic and the changes they could implement, aside from or in addition to the main institution. ^+^ Below 50%: Begin a focused health literacy initiative to eliminate literacy-related barriers in this area. 50% to 75%: Augment efforts to eliminate literacy-related barriers in this area. 76% to 85%: Continue to augment efforts; monitor and document changes.

## Data Availability

The data are all provided in this article.
